# Sexual performance and semen quality of pubertal lambs treated with different weaning methods

**DOI:** 10.5194/aab-65-259-2022

**Published:** 2022-07-20

**Authors:** Rogelio Alejandro Ledezma-Torres, Fernando Sánchez-Dávila, Diana Aimé Rodríguez-Miranda, Carlos Luna-Palomera, Juraj Grizelj, José Fernando Vázquez-Armijo, Nicolás López-Villalobos

**Affiliations:** 1 Universidad Autónoma de Nuevo León, Facultad de Medicina Veterinaria y Zootecnia, Posgrado Conjunto FA-FMVZ, General Escobedo, CP 66050, Mexico; 2 Universidad Autónoma de Nuevo León, Facultad de Agronomía, Posgrado Conjunto FA-FMVZ, Laboratorio de Reproducción Animal, Unidad Académica Marín, Marín, CP 66700, Mexico; 3 Universidad Autónoma de Nuevo León, Posgrado Conjunto FA-FMVZ, General Escobedo, CP 66050, Mexico; 4 Universidad Juárez Autónoma de Tabasco, División Académica de Ciencias Agropecuarias, Villahermosa, Tabasco, CP 86280, México; 5 Universidad de Zagreb, Facultad de Medicina Veterinaria, Zagreb, Croatia; 6 Universidad Autónoma del Estado de México, Centro Universitario Temascaltepec, Temascaltepec, CP 51300, Mexico; 7 School of Agriculture and Environment, Massey University, Palmerston North 4442, New Zealand

## Abstract

The objective of this study was to determine the effect of the weaning method on lamb stress, body weight, sexual behavior, and semen quality of Saint Croix male lambs. The present study was carried out during the late spring and summer of 2018 in the northeast of Mexico. Sixty male lambs born as twins or triplets (3.2 ± 0.6 kg birth weight) and weaned at 60 d of age (19.21 ± 1.8 kg weaning weight) were divided into two weaning methods: complete separation from the dams (CS; the lambs were moved to a pen that was at 500 m of distance from the dams) and separation with contact from the dams (SCD); the lambs were physically separated by a steel mesh that prevented the lambs from having the possibility of sucking milk from their mothers, but they maintained permanent visual and auditory contact. Cortisol levels were determined 3 d before and 7 d after weaning. Lambs were evaluated as 3-month-old lambs for sexual behavior and semen quality for 9 weeks. The effects of the weaning method (M), week (W), and the interaction M × W were significant on body weight and cortisol levels ( P<0.001 ). The SCD lambs had higher cortisol levels at 3, 5, and 7 d after weaning than CS lambs ( P<0.001 ). The CS lambs had higher body weight during the first 4 weeks after weaning than SCD lambs ( P<0.001 ). The weaning method had no effect on scrotal circumference, sexual behavior, and semen quality traits, except for progressive sperm motility, being better for the lambs that were completely separated ( P<0.05 ). The results from this study show that complete separation of lambs and ewes at weaning is an effective method to reduce lamb stress and improve lamb growth after weaning, but it did not have long-term effects on sexual behavior and semen quality of Saint Croix male lambs.

## 1 Introduction

The Saint Croix sheep is one of the hair breeds originating from the Virgin Islands. The ewes are prolific and show low or no seasonality for breeding (Sánchez Dávila et al., 2015). Due to these characteristics, this breed is exploited in the northeast of Mexico as a maternal line achieving parturition every 8 months (Dávila et al., 2011). Weaning is a stressful event for the lamb because maternal milk is replaced by solid food and the mother–lamb bond is broken (Orihuela et al., 2004; Napolitano et al., 2008; Wang et al., 2019). Stress caused at weaning can negatively impact the overall health and production of the lamb as shown in decreased growth rates and food intake (Schichowski et al., 2008; Pascual-Alonso et al., 2015; Barnard et al., 2016), changes in physiologic, endocrine and immune responses, and increased susceptibility to disease and infection (Orgeur et al., 1999; Backes et al., 2015; Destrez et al., 2017) manifested in the post-weaning period. Effect of stress at weaning on future reproductive performance of ram lambs has not been reported, but other factors such as maternal stress during late pregnancy (Henrique et al., 2020) and birth season (Sánchez-Dávila et al., 2019) have been reported to influence post-weaning sperm quality and sexual development of ram lambs.

In this study, two methods of lamb weaning have been proposed: complete (CS) and separation with contact from the dams (SCD). In CS the lambs are placed in pens that are completely separated from the ewes with no contact. In SCD the lambs are placed in adjacent pens for several weeks where they cannot suckle milk from the mother, but the social–affective contact between lamb and mother continues (Freitas-de-Melo and Ungerfeld, 2016). The study by Sowinska et al. (2001) showed that temporary separation before weaning from 15 d of age improved the health and immune status of the lamb by supplementing with pre- and post-weaning probiotics (Viérin and Bouissou, 2003; Kumar et al., 2014). It is unknown if SCD has a prolonged effect on stress levels or has a negative effect on post-weaning productive performance of lamb (Teixeira et al., 2014; Backes et al., 2015). Similarly, the possible effects on their sexual behavior and post-weaning semen quality are unknown. The objective of the present study was to determine the effects of the weaning method on lamb stress measured using cortisol levels at weaning, growth performance from weaning (2 months) to 5 months, and sexual behavior and semen quality of 3-month-old Saint Croix ram lambs. The research hypothesis was that lambs that were completely separated from their mothers will have better reproductive development during the growth stage by presenting better body development after weaning.

## 2 Materials and methods

### 2.1 Location of the study

The research was carried out from May to August 2018 using animals from the Saint Croix sheep flock of the Facultad de Agronomía and the Laboratorio de Reproducción Animal of the Universidad Autonoma de Nuevo León (UANL), located in Marín, Nuevo León, Mexico, at the coordinates 25 ${}^{\circ }$ 50 ${}^{\prime }$ 34 ${}^{\prime \prime }$ north latitude and 100 ${}^{\circ }$ 04 ${}^{\prime }$ 21 ${}^{\prime \prime }$ west longitude and at an altitude of 333 m. Temperatures vary from 18 to 43 ${}^{\circ }$ C in summer and from 10 to - 2 ${}^{\circ }$ C in winter. The average annual temperature is 23.1 ${}^{\circ }$ C with an average annual rainfall of 429 mm. During the study, the average ambient temperatures that prevailed were 32–35 ${}^{\circ }$ C with a minimum of 23 ${}^{\circ }$ C and a maximum of 38 ${}^{\circ }$ C. The number of light hours that occurred during the study was 13.5 ± 0.32 h.

### 2.2 Animals

Sixty Saint Croix lambs born as twins or triplets in the spring of 2018 with an average birth weight of 3.2 ± 0.6 kg (mean ± SD) were selected. The dams of the lambs came from the same flock, where they grazed buffel grass (*Cenchrus ciliaris*) pastures and received a commercial concentrate that contained 12 % crude protein and 2.5 Mcal kg ${}^{-1}$ dry matter (DM). During the lactation period the lambs remained in the pens while the dams grazed for 8 h a day and received a pelleted concentrate containing 21 % crude protein and 2.0 Mcal kg ${}^{-1}$ DM.

### 2.3 First stage

**Figure 1:**
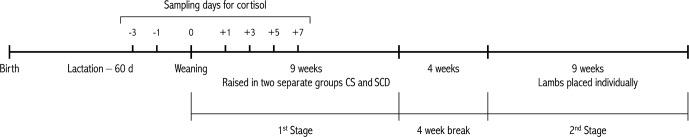
Study plan.

The lambs were weaned at 60 d of age following two methods: complete (CS) and separation with contact from the dams (SCD) (Fig. 1). In the group of lambs that were completely separated from the ewes (initial weight = 13.2 ± 1.61 kg) (mean ± SD) the lambs were moved to a pen that was at 500 m of distance from the dams, where they could not hear, see, or smell their mothers. In the group of lambs that were partially separated from the ewes (initial weight = 14.4 ± 2.2 kg) the lambs were physically separated by a steel mesh that prevented the lambs from having the possibility of sucking milk from their mothers, but they maintained permanent visual and auditory contact. Both groups of lambs were housed separately in 30 m ${}^{2}$ pens. The feeding of the lambs with a solid meal began from 15 d of age until weaning, supplying a freely accessible concentrate containing 18 % crude protein and 2.1 Mcal of metabolizable energy per kilogram DM.

Figure 1 presents a schematic representation of the experiment in two stages according to the age of the lambs. The first stage was from the weaning of the lambs up to 16 weeks of age. The second stage started when the lambs were on average 20 weeks old, and the purpose of this stage was to measure the sexual behavior and semen quality of the ram lambs for 9 weeks.

#### 2.3.1 Cortisol determination

For a period of 1 week, blood samples were taken for the determination of cortisol at days - 3, - 1, 0, 3, 5 and 7 considering the day of weaning as 0. The blood sample was taken by jugular vein puncture, using tubes without anticoagulant. Samples were taken at 06:00 and the lambs were fasting. The blood samples were centrifuged at 1500×g for 20 min at 19 ${}^{\circ }$ C to separate the serum, which was stored at - 20 ${}^{\circ }$ C for further analysis. The samples were analyzed using a commercial Cortisol kit (Mex-Lab, Jalisco, Mexico), which have 7 and 2100 nmol L ${}^{-1}$ of minimum and maximum cortisol sensitivity.

#### 2.3.2 Body weight

After weaning, nine weekly measures of body weight were recorded from each lamb using an automatic weight scale (Gallagher W210, New Zealand).

### 2.4 Second stage

Four weeks after stage 1 finished, when the male lambs were about 5 months old, each lamb was placed individually in a pen of 2 m ${}^{2}$ and kept for a period of 9 weeks with freely accessible feed with a concentrate containing 16 % of crude protein and 2.2 Mcal kg ${}^{-1}$ (Fig. 1).

#### 2.4.1 Body weight

Nine weekly measures of body weight were recorded from each lamb using an automatic weight scale (Gallagher W210, New Zealand).

#### 2.4.2 Scrotal circumference

The scrotal circumference was measured with a metallic band (Nasco, Wisconsin, USA) in the middle part, causing both testes to be pulled to the bottom of the scrotal sac.

#### 2.4.3 Sexual behavior

The sexual behavior of each lamb was evaluated weekly using two ewes of the same breed estrogenized with 1 mg of estradiol benzoate (Syntex, Virbac, Jalisco, Mexico) 2 d before the behavior test. The lambs were individually exposed to a ewe for a period of 20 min in a 3 m ${}^{2}$ pen without the rest of the lambs observing the sexual activity of each one of them. The variables that were evaluated were mounts ( M ), anogenital sniffing (AS), lateral approaches (LA), flehmen ( F ), attempts to mount (AM), and mounts with ejaculation (ME).

#### 2.4.4 Semen quality

The quality of the semen of each lamb was evaluated weekly. The ejaculate was obtained by means of an electro-ejaculator (21160, Bailey^®^, Colorado, USA). The electro-ejaculator probe was inserted rectally into each lamb and stimulated with electrical impulses of 2 V for 2–3 s each, with a rest for 2 s until a semen sample was extracted, and immediately evaluated for the following characteristics: volume (mL) mass motility (1–5), progressive motility (0 %–100 %), and sperm concentration (millions/mL ejaculate).

### 2.5 Statistical analysis

The data were analyzed separately for each stage. Repeated measures of body weight, scrotal circumference, and characteristics of sexual behavior and semen quality on the same animal were analyzed with a linear model that included the fixed effect of weaning method (CS and SCD), week of measure, and the interaction between weaning method and week. The model for the analysis of variance of cortisol levels was the same, but day of the measure was included instead of a week of measure, and the random effect of lamb inside treatment was considered. Least squares means for each level of these effects were obtained, and the difference between means of two treatments (CS vs. SCD) was obtained by orthogonal contrast by Proc Mixed of SAS. Significant differences between the means were declared at P<0.05 . Statistical analyses were performed using SAS version 9.0 (SAS Institute Inc.).

## 3 Results

The effects of the weaning method, time, and their interaction on the traits studied at both stages are presented in Table 1. The effects of the weaning method, week, and interaction between weaning method and week (or day in the case of cortisol level) were all significant on body weight and cortisol levels ( P<0.001 ), but not on scrotal circumference. In general, the effect of the weaning method was not significant on scrotal circumference, sexual behavior, and semen quality traits, except for progressive sperm motility ( P<0.05 ). Effect of the week was significant ( P<0.005 ) on body weight, mass sperm motility, progressive sperm motility, and all sexual behavior traits, except for flehmen.

**Table 1:** Effects of weaning method and time of measure on cortisol levels after weaning, scrotal circumference and growth, and sexual behavior traits of Saint Croix lambs.

		Weaning method ${}^{1}$		P value		
Traits		Complete	SCD ${}^{2}$	Method (M)	Week (W)	Interaction M × W
Stage 1: immediately after weaning	Mean ( ± SE)	Mean ( ± SE)	Mean ( ± SE)			
Body weight (kg)	19.05 ± 0.17	20.17 ± 0.21	17.95 ± 0.25	0.001	0.001	0.001
Scrotal circumference (cm)	17.13 ± 0.40	16.87 ± 0.40	17.23 ± 0.30	ns	ns	ns
Cortisol (nmol L ${}^{-1}$ )	65.09 ± 3.65	48.47 ± 3.77	81.70 ± 3.77	0.001	0.001 ${}^{*}$	0.001 ${}^{*}$
Stage 2: age 4–6 months						
Body weight (kg)	34.2 ± 0.37	34.7 ± 0.5	33.7 ± 0.5	ns	0.001	ns
Scrotal circumference (cm)	27.62 ± 0.23	27.3 ± 0.3	27.9 ± 0.3	ns	ns	ns
Semen volume (mL)	0.67 ± 0.24	0.69 ± 0.2	0.64 ± 0.2	ns	ns	ns
Mass sperm motility	1.35 ± 0.09	1.5 ± 0.1	1.2 ± 0.1	ns	0.001	ns
Progressive sperm motility (%)	50.67 ± 5.52	51.3 ± 3.0	50.3 ± 2.9	0.050	0.001	ns
Sperm count per mL of collected semen ( $\times 10^{7}$ )	1.16 ± 0.14	1.26 ± 0.2	1.10 ± 0.2	ns	ns	ns
Reaction time (s)	4.02 ± 0.39	3.6 ± 0.5	4.4 ± 0.6	ns	0.002	ns
Anogenital sniffing	5.2 ± 0.29	5.6 ± 0.4	4.9 ± 0.6	ns	0.001	0.01
Flehmen	1.87 ± 0.21	2.0 ± 0.3	1.9 ± 0.3	ns	ns	ns
Lateral approaches	14.4 ± 0.92	14.4 ± 1.3	12.9 ± 1.3	ns	0.001	ns
Mounts attempts	6.32 ± 0.48	6.0 ± 0.7	6.6 ± 0.7	ns	0.005	ns
Number of mounts	2.17 ± 0.26	2.3 ± 0.4	2.0 ± 0.4	ns	0.001	ns
Number of mounts with ejaculation	1.0 ± 0.10	1.1 ± 0.1	1.0 ± 0.2	ns	0.030	ns

${}^{1}$ The lambs were weaned on average at 60 d of age following two methods: complete separation in a pen located 500 m of distance from the dams and partial separation in a pen separated with a steel mesh from the pen of dams. ${}^{2}$ Separation with contact from the dams (SCD). ${}^{*}$ This is the P value for effect of day and interaction M × W d. ns: not significant.

**Figure 2:**
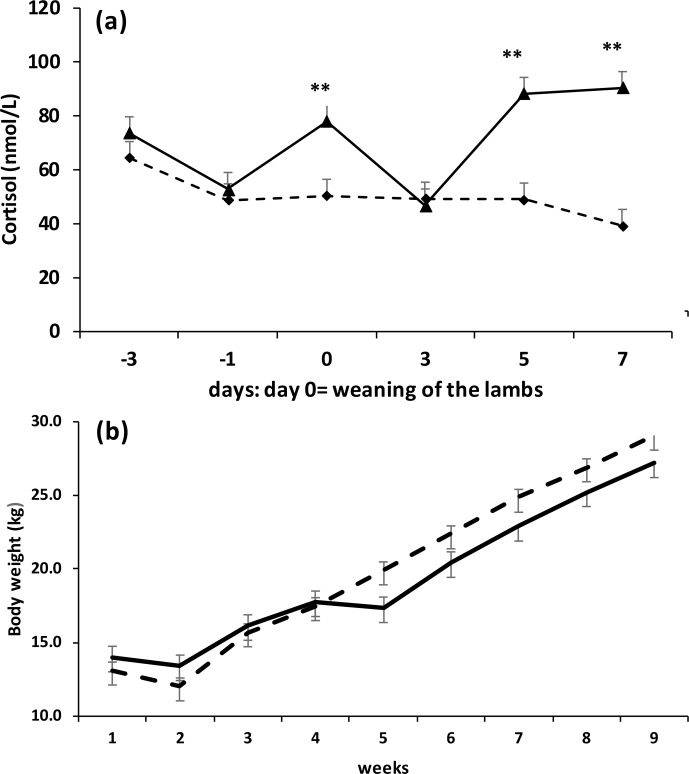
Cortisol levels **(a)** and body weight **(b)** in Saint Croix lambs after weaning on average at 60 d of age following two methods: complete separation (dotted line) where the lambs were placed in a pen located 500 m of distance from the dams and separation with contact from the dams (black line) where the lambs were placed in a pen separated with a steel mesh from the pen of dams ( P<0.05 ).

Compared to CS lambs, the SCD lambs had higher ( P<0.001 ) cortisol levels for 9 consecutive days, where they maintained visual and auditory contact with their dams, but no access to milk (Fig. 2a). The CS lambs were heavier ( P<0.001 ) at week 4 after weaning than the SCD lambs, and this difference was maintained for 9 weeks (Fig. 2b).

## 4 Discussion

In the present study, the objective was to determine two weaning methods (CS and SCD) in hair lambs: completely separated vs. separated with contact from their dams. In the first stage, the effect of type of weaning on body development and cortisol levels was evaluated, and in the second stage the effect of type of weaning on sexual behavior and seminal quality was evaluated. It was observed that in the first stage the lambs that were completely separated from their mothers presented better body development; however, in the second stage, there were no differences between the two types of weaning in terms of sexual behavior and seminal quality. The results showed that the SCD lambs were more stressed and were lighter than the CS lambs. In the opposite direction of the findings, Godfrey et al. (2016) reported that lambs weaned abruptly at an age younger than 60 d showed high cortisol levels as the result of stress, which was manifested as marked vocalization and agitation. In our study, the SCD lambs observed and listened to their mothers during the entire post-weaning process, which causes the stress level to rise with the consequence of the presence of diseases (Destrez et al., 2017). Schichowski et al. (2008) reported that lambs that were completely separated from the dams complemented the lack of milk with the meal offered by dedicating more time for eating, which is reflected in better body development. On the other hand, lambs weaned at an age of 90 d show lower cortisol levels, manifesting in lower stress (Sowinska et al., 2001). It has been reported that age at weaning plays an important role in the reproductive development of young bucks. Amjad et al. (2021) found better testicular development and more testosterone release in Beetal young bucks weaned at 16 weeks of age, compared to young bucks weaned at 8 weeks of age.

The CS weaning method evaluated in this study resulted in lambs that were less stressed than the SCD lambs; however, this did not affect sexual behavior and semen quality when the animals were evaluated as 3-month-old ram lambs. Other factors, such as feeding (Mekoya et al., 2009) and rearing (Santos et al., 2015) systems, may have larger effects on the sexual development of male rams than stress at weaning. For example, Mekoya et al. (2009) found that the inclusion of *Sesbania sesban* (a fodder tree) up to 30 % of the ratio improved feed intake, growth rate, onset of puberty, and sexual development of male and female Menz lambs. Santos et al. (2015) found that ram lambs reared individually had better sexual development, compared to ram lambs reared in a group. In the current study, lambs were weaned at 60 d, and the body weight of CS lambs was higher compared with SCD lambs. This could be due to the fact that, by not facing distractions due to the presence of the mother, the lambs dedicated themselves to consuming food and their blood metabolites could quickly stabilize without causing important physiological disorders at the rumen level (Mora-Medina et al., 2017), thereby achieving better live weights. It is also important to mention that both groups of lambs presented acceptable body development because after weaning they were fed with a concentrate that covered their nutritional requirements for maintenance, activity, and growth. This feeding strategy sometimes does not happen in commercial flocks: in many cases the lambs are sent to pastures consuming native pastures and fodders, and their body development is negatively affected and they become prone to internal parasite infections (Karakus, 2014).

The stress caused to the lamb at weaning can be increased if the lamb is in poor body condition (Chai et al., 2015), exposed to parasite infestation (Campbell et al., 2017), or with health problems (Destrez et al., 2017). While these changes in lamb body development have been shown to occur in the first-week post-weaning, many stressors can occur, including social, environmental, physical, and nutritional factors (Alves et al., 2016), which affect performance post-weaning (Henrique et al., 2018). However, one of the objectives of our study was to evaluate the effect of weaning method on the sexual behavior and semen quality of the 3-month-old rams. This is contrary to Damián et al. (2017), who found that the effect of the dam plays a preponderant role during the rearing of lambs, affecting sexual behavior and testosterone levels of the ram during adulthood, compared to the lambs that were separated from their mothers between 24 and 36 h after birth. It is important to mention that the stress was caused by the complete separation of the lambs from the ewes in both times after birth; not only cortisol levels could be increased, but other important metabolites such as glucose, lactate, CO ${}_{2}$ , O ${}_{2}$ , among others, could also be altered (Mora-Medina et al., 2017), which may present greater physiological disturbances, compromising the health of the lambs at the time of weaning. However, the aforementioned metabolites are restored more quickly when the lambs are older (60 d of weaning), which is the case for the lambs in our study. This may be the reason that the sexual behavior and semen quality were similar for both weaning methods, suggesting that there may be other factors that affect the reproductive performance of ram lambs. For example, Henrique et al. (2018) found that malnutrition of the ewe in the last third of pregnancy affected the testicular weight of the lambs, and that this effect was greater when accompanied by abrupt weaning of the lambs (Henrique et al., 2017).

The only trait related to semen quality that was different between weaning methods was progressive sperm motility, which was superior for the CS lambs. This difference may be explained by an early intake of fibrous components in the diet that favored the development of the rumen (Urbano et al., 2017), which in turn could increase the assimilation of nutrients that favored the development of this characteristic in the ejaculate (Blache and Martin, 2009). Another possible explanation for not finding significant effects of the weaning method on sexual behavior and semen quality is that regardless of the weaning method, male lambs are less stressed than females (Freitas-de-Melo and Ungerfled, 2020), in such a way that other possible factors are involved in this reproductive process (Sánchez-Dávila et al., 2019). For example, Saint Croix lambs are reported to be sexually precocious (Wheaton and Godfrey, 2003) and adapted to different environments; therefore, it is considered that although the SCD lambs were more stressed, and developed more slowly, this was not an impediment for them to develop sexually in a similar way to the CS lambs. It is also important to note that the second stage of this study was carried out at the start of the natural breeding season (June–August) when lambs may express higher sexual activity. The study carried out by Sánchez et al. (2019) showed that the reproductive performance of Saint Croix male lambs born in spring was better than that of lambs born in summer and autumn. The results reported by Santos et al. (2015) and Sanchez et al. (2019) suggest that there is a significant effect of an interaction between birth season and weaning method on the sexual behavior and semen quality of male lambs, and this should be a matter of future research.

## 5 Conclusions

The results from this study show that complete separation of lambs and ewes at weaning at 2 months of age decreases stress and improves body weight of Saint Croix male lambs, but their sexual behavior and semen quality as ram lambs are not affected. A practical application of these results is that complete separation of the lambs would be the recommended weaning method when the lambs are to be fattened since the post-weaning growth rate could be increased.

## Data Availability

The original data are available upon request to the corresponding author.
